# A Prospective Cohort Study to Examine the Association between Dietary Patterns and Depressive Symptoms in Older Chinese People in Hong Kong

**DOI:** 10.1371/journal.pone.0105760

**Published:** 2014-08-22

**Authors:** Ruth Chan, Dicken Chan, Jean Woo

**Affiliations:** 1 Department of Medicine and Therapeutics, The Chinese University of Hong Kong, Shatin, Hong Kong; 2 The Jockey Club School of Public Health and Primary Care, The Chinese University of Hong Kong, Shatin, Hong Kong; Institute of Psychiatry, United Kingdom

## Abstract

**Introduction:**

Dietary patterns are culturally specific and there is limited data on the association of dietary patterns with late-life depression in Chinese. This study examined the associations between dietary patterns and baseline and subsequent depressive symptoms in community-dwelling Chinese older people in Hong Kong.

**Methods:**

Participants aged ≥65 year participating in a cohort study examining the risk factors for osteoporosis completed a validated food frequency questionnaire at baseline between 2001 and 2003. Factor analysis was used to identify three dietary patterns: “vegetables-fruits” pattern, “snacks-drinks-milk products” pattern, and “meat-fish” pattern. Depressive symptoms were measured at baseline and 4-year using the validated Geriatric Depression Scale. Multiple logistic regression was used for cross-sectional analysis (n = 2,902) to assess the associations between dietary patterns and the presence of depressive symptoms, and for longitudinal analysis (n = 2,211) on their associations with 4-year depressive symptoms, with adjustment for socio-demographic and lifestyle factors.

**Results:**

The highest quartile of “vegetables-fruits” pattern score was associated with reduced likelihood of depressive symptoms [Adjusted OR = 0.55 (95% CI: 0.36–0.83), *p_trend_* = 0.017] compared to the lowest quartile at baseline. Similar inverse trend was observed for the highest quartile of “snacks-drinks-milk products” pattern score [Adjusted OR = 0.41 (95% CI: 0.26–0.65), *p_trend_*<0.001] compared to the lowest quartile. There was no association of “meat-fish” pattern with the presence of depressive symptoms at baseline. None of the dietary patterns were associated with subsequent depressive symptoms at 4-year.

**Conclusion:**

Higher “vegetables-fruits” and “snacks-drinks-milk products” pattern scores were associated with reduced likelihood of baseline depressive symptoms in Chinese older people in Hong Kong. The longitudinal analyses failed to show any causal relationship between dietary patterns and depressive symptoms in this population.

## Introduction

Depression is a common mental health disorder. It affects over 350 million people worldwide and it is expected that depression will become the world's leading cause of disease burden by the year 2030 [1]. Depression is less prevalent among older adults than among younger counterparts [2]. However, late-life depression is considered as an important public health problem due to its devastating consequences, including increased risk of morbidity and reduced physical, cognitive, and social functioning [3], [4]. As a result, to identify modifiable risk factors for depression is inevitably important [5].

Recent evidence suggests that diet modifies key biological factors associated with the development of depression, and diet rich in fruits, vegetables, whole grains and fish is in general associated with lower risk of depression [6], [7]. Studies have shown that the antioxidant compounds in fruit and vegetables could reduce neuronal damage induced by oxidative-stress whereas long-chain omega-3 polyunsaturated fatty acids could possibly affect mood by an alteration of the brain serotonergic function and its immune-neuroendocrine effects [6], [7]. However, the examination of diet as a risk factor for depression has traditionally focused on the effect of single foods and nutrients, and the findings thus far are inconsistent [8]. Since diet is a combination of food and nutrients, there has been an increasing interest in using dietary pattern analysis in epidemiological studies [9]. There are two main approaches to define dietary patterns [9], [10]. One approach is the *a priori* approach in which dietary indices are constructed based on prevailing dietary recommendations. Another approach is the *a posteriori* approach in which dietary patterns are derived from statistical modeling, such as factor analysis, using data from dietary records or food frequency questionnaires (FFQ).

Data on the association between dietary patterns and depression are limited. Some cross-sectional studies have been carried out in Caucasians and Japanese to evaluate the association between dietary patterns and depression or depressive symptoms in adulthood [11]–[16]. To our knowledge, nine prospective studies have examined the role of dietary patterns and depression or depressive symptoms in adulthood [17]–[25] and two prospective studies have evaluated this association in adolescents [26], [27]. Few studies have investigated the association between depression and diet in Chinese older people, and these studies mainly focused on the effect of single foods and nutrients [28], [29]. Only one cross-sectional study has examined the association of dietary patterns with depressive symptoms in Chinese but it was done in adolescents [27].

Since dietary patterns are culturally specific and there is limited data on the association of dietary patterns with late-life depression in Chinese, we evaluated the association between dietary patterns and baseline and subsequent depressive symptoms in older Chinese people, using data from a sample of community-dwelling men and women aged 65 and over participating in a prospective study in Hong Kong. We hypothesized that dietary patterns are associated with baseline and subsequent depressive symptoms in this population.

## Methods

### Study population

Subjects were participants of a cohort study examining the risk factors for osteoporosis in Hong Kong [30]. 2,000 men and 2,000 women aged 65 years and over living in the community were recruited in a health survey between August 2001 and December 2003 by recruitment notices and talks in community centers and housing estates. Participants were volunteers and were able to walk or take public transport to the study site. They were recruited using a stratified sample so that approximately 33% would be in each of these age groups: 65–69, 70–74, 75+. Compared with the official population statistics, participants had higher educational level than the overall older population in Hong Kong (12–18% vs. 3–9% with tertiary education in the age groups 80+, 75–79,70–74, and 65–69 years) [31]. The 4-year follow-up was held between August 2005 and November 2007. Follow-up was done by a mailed reminder for a follow-up body check appointment. Phone reminders were given again close to the appointment dates, and defaulters were given a second appointment to enhance attendance rates. Mean (SD) follow-up year was 3.9 (0.1) years.

**Figure 1:**
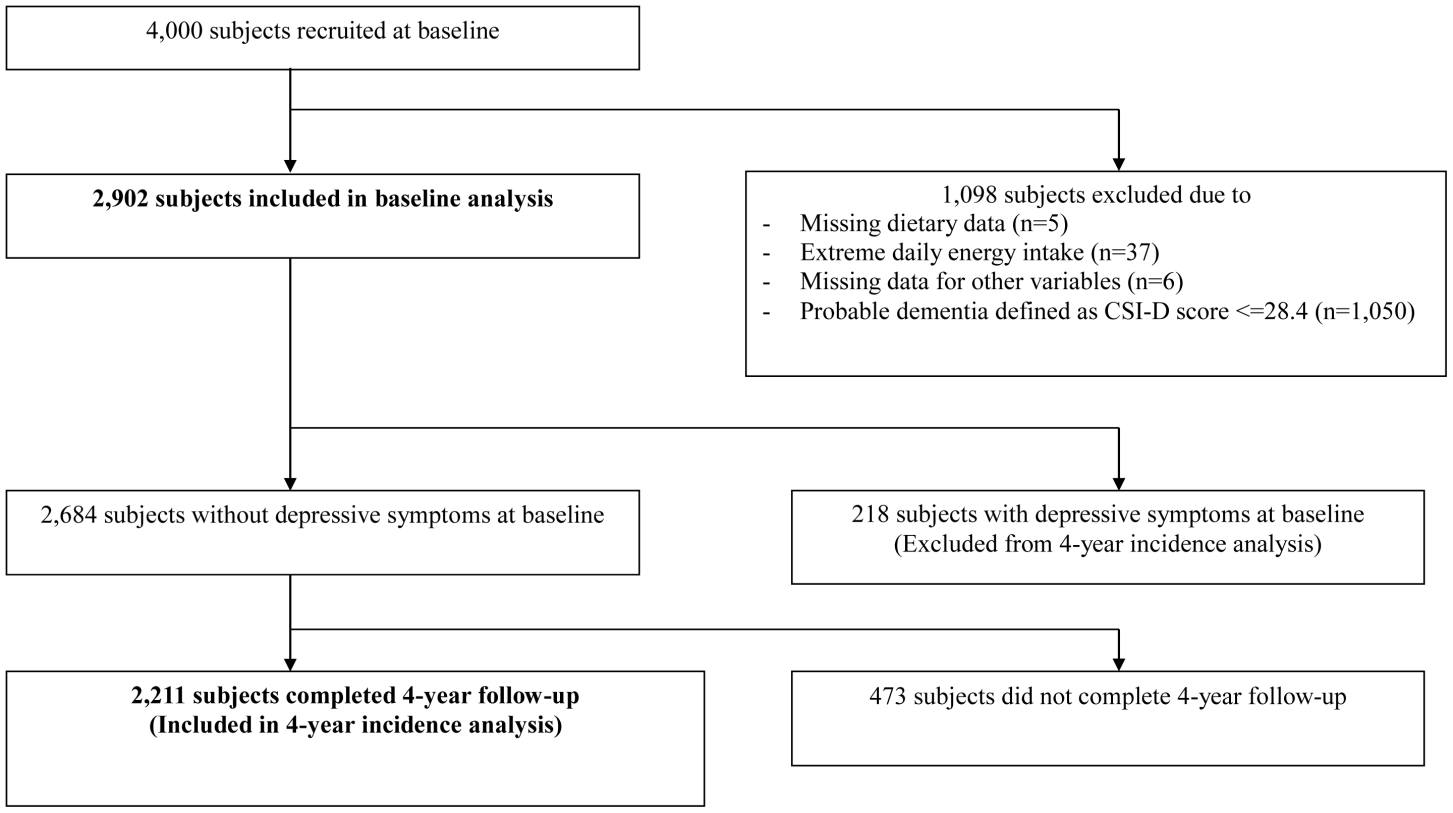
Number of subjects included and excluded for baseline and 4-year follow-up analyses.

We excluded participants who did not have dietary data (n = 5), those with extreme daily energy intake at the first- and last-half percentiles of the sex-specific range (n = 37), those who had missing data for the variables included for the analyses (n = 6), and those who were probable dementia defined using the cognitive part of the Community Screening Instrument for Dementia (CSI-D) with the cutoff point of ≤28.4 [32] (n = 1,050). The baseline analysis was performed on 2,902 participants. For the longitudinal analysis, we further excluded 218 participants who had depressive symptoms at baseline and 473 participants who did not attend the 4-year follow-up. 2,211 participants were therefore included for the 4-year incidence analysis (Figure 1). This study was conducted in accordance with the Declaration of Helsinki. This study was approved by the Clinical Research Ethics Committee of the Chinese University of Hong Kong. Written informed consent was obtained from all participants.

### Demographic and overall health characteristics

A standardized interview was performed to collect information on age, gender, education level, marital status, smoking habit, alcohol use and medical history. Information on the duration and level of past and current use of cigarettes, cigars and pipes was obtained. Smoking history was classified in terms of former smoking (at least 100 cigarettes smoked in a lifetime), current smoking and never smoking. Drinking status was defined as never, former (ever drank at least 5 drinks daily in a lifetime) or current drinker. Baseline disease status was obtained by self-report of their doctors' diagnoses, supplemented by the identification of drugs brought to the interviewers.

### Anthropometric data

Body weight was measured to the nearest 0.1 kg with participants wearing a light gown, using the Physician Balance Beam Scale (Healthometer, Illinois, USA). Height was measured to the nearest 0.1 cm using the Holtain Harpenden stadiometer (Holtain Ltd, Crosswell, UK). Body mass index (BMI) was calculated as body weight in kg / (height in m)^2^.

### Physical activity assessment

Physical activity was assessed by the Physical Activity Scale for the Elderly (PASE) [33], which was adapted for use in elderly Hong Kong Chinese [34]. This is a 12-item scale measuring the average number of hours per day spent in leisure, household, and occupational physical activities over the previous 7-day period. Activity weights for each item were determined based on the amount of energy spent, and each item score was calculated by multiplying the activity weight with daily activity frequency. A composite PASE score of all the items was yielded. A higher PASE score reflects higher physical activity level.

Number of instrumental activities of daily living (IADLs) were assessed using specific questions and participants were asked to self-report of any impairment in walking two to three blocks outdoors on level ground, climbing 10 steps without resting, preparing own meals, doing heavy housework like scrubbing floors or washing windows, and shopping for groceries or clothes. A summed score from 0 to 5 was calculated from these activities as the degree of impairment in IADLs, with higher score indicating greater impairment.

### Assessment of cognitive function

Since depression may be associated with cognitive impairment, in the analysis of factors associated with depression, the presence of cognitive impairment was adjusted for as a confounding factor. Cognitive function was assessed by trained research staff using the cognitive part of the Community Screening Instrument for Dementia (CSI-D) [32], validated in different cultural and educational settings [32]. The cognitive part of the CSI-D consists of 32 items in six cognitive domains: orientation to time, orientation to place, praxis, abstract thinking, language and memory. A summary score ranged from 0 to 33 was generated, with higher score meaning better cognitive function. The cutoff point for probable dementia is ≤28.4.

### Assessment of depressive symptoms

Depressive symptoms was assessed by face-to-face interviews using a validated Chinese version of the Geriatric Depression Scale (GDS) [35], [36]. The GDS short form was found to be a highly reliable (reliability coefficient of 0.90) and valid screening device (sensitivity of 96.3% and specificity of 87.5%) for assessing geriatric depression in Hong Kong Chinese [36]. The GDS short form consists of 15 questions relevant to depression, such as motivation, self-image, losses, agitation and mood. A yes/no format was designed for each question. A summary score ranged from 0 to 15 was generated, and a cut-off of 8 or more was used to define the presence of depressive symptoms.

### Dietary assessment

Baseline dietary intake was assessed using a validated FFQ developed in a population survey [37]. Daily nutrient intake was calculated using food tables derived from McCance and Widdowson [38] and the Chinese Medical Sciences Institute [39]. The FFQ consisted of 280 food items. Each participant reported the frequency and the usual amount of consumption of each food item over the past year. For each food 9 different categories were given from never or seldom to more than once a day. Portion size was explained to participants using a catalogue of pictures of individual food portions. For seasonally consumed vegetables and fruits, participants were asked about the months of food consumption over the past year. The amount of cooking oil was estimated according to the usual cooking methods of preparing standardized portion of different foods and the usual portion of different foods consumed by the participants.

### Dietary patterns derived by factor analysis

Details of dietary pattern scores derived by the factor analysis have been described elsewhere [40]. In brief, individual food items from the FFQ were aggregated into 32 food groups based on similarity of type of food and nutrient composition. The food groups were energy adjusted by dividing the energy intake from each food group by total energy intake and multiplying by 100, and were expressed as percentage contribution to total energy [41]. Factor analysis was conducted with varimax rotation using the 32 food groups [9]. Factors were retained based on an eigenvalues greater than 1.0, a scree plot, and the interpretability [42]. The factor scores for each pattern were calculated for each participant by summing intakes of food items weighted by their factor loadings. A higher score indicated greater conformity with the pattern being calculated.

### Statistical analysis

Statistical analyses were performed using the statistical package SPSS version 21.0 (SPSS Inc., Illinois, US). Data were checked for normality using histograms and logarithmic transformation was applied whenever appropriate. Dietary pattern scores derived by factor analysis were stratified into quartiles based on the distribution of each sex. Pearson's correlation was used to examine the correlation between each dietary pattern score and intakes of various nutrients. Independent *t* test and chi square test were used to examine baseline differences in mean age, BMI, PASE, CSI-D score, energy intake, and also differences in the distribution of sex, education level, smoking status, alcohol use, number of IADLs, self-reported disease status, quartiles of each dietary pattern score between participants with depressive symptoms and participants without depressive symptoms at baseline. These tests were also used to examine differences in baseline characteristics between participants who included and excluded in the baseline analysis, and between participants who completed and participants who did not complete the 4-year follow-up.

Multivariate logistic regression was used to estimate the odds ratio (OR) and 95% confidence intervals (CIs) for both the baseline and the subsequent depressive symptoms according to quartiles of each dietary pattern score. Model 1 was adjusted for baseline age (years), sex, and daily energy intake (kcal). Model 2 was further adjusted for baseline BMI, PASE, number of IADLs (no difficulty/some difficulties), smoking habit (never/past/current), alcohol use (never/past/current), education level (no education/primary or below/secondary or matriculation/University or above), and marital status (married or living with a partner/widowed, separated or divorced/single). Model 3 was further adjusted for baseline self-reported history of diabetes (yes/no), hypertension (yes/no), and CVD or stroke (yes/no), and CSI-D score. Test for trend was examined by entering quartiles of each dietary pattern score in all models. An α level of 5% was used as the level of significance. Interaction between sex and quartiles of each dietary pattern score was tested by addition of cross-product terms to the multivariate models. Interactions were not significant, thus all analyses are presented in the total population.

To check the possibility of reverse causation, a sensitivity analysis excluding participants with extreme GDS at the top two percentiles (GDS≥11) was performed for the cross-sectional data. Considering that GDS is a screening scale and may be limited as a case-defining instrument compared to other diagnostic instruments [43], multivariate logistic regression models were repeated using a group whose depressive symptom levels had clearly increased over 4-year (i.e. with an increase in GDS score at 4 and over from baseline to four years) as an outcome variable. This cut-off value was chosen because 142 participants (6.4%) could be classified as the worsening cases and this number of cases may be more sufficient and powerful for statistical analysis than using an increase in GDS score at 5 and over as a cut-off value (i.e. n = 85). All models were adjusted for the same variables mentioned above.

## Results

### Dietary patterns of the participants

Factor analysis identified three dietary patterns (Table 1). The first factor (vegetables-fruits pattern) was dominated by frequent intake of vegetables, fruits, soy and soy products, and legumes. The second factor (snacks-drinks-milk products pattern) was composed of a mixture of healthy and unhealthy food groups. It was characterized by frequent intake of condiments, drinks, fast food, French fries, potato chips, sweets and desserts, nuts, milk products, and whole grains. The third factor (meat-fish pattern) included frequent intake of dim sum, red and processed meats, poultry, fish and seafood, and wine.

**Table 1:** Food group factor loading^a^ for three dietary patterns.

Food groups	Dietary patterns		
	Factor 1: Vegetables-fruits	Factor 2: Snacks-drinks-milk products	Factor 3: Meat-fish
Other vegetables	**0.60**	-0.08	0.03
Cruciferous vegetables	**0.48**	−0.08	−0.06
Tomatoes	**0.47**	0.05	−0.04
Soy	**0.43**	0.03	0.10
Dark green & leafy vegetables	**0.42**	−0.27	−0.04
Starchy vegetables	**0.42**	0.07	−0.03
Fruits	**0.39**	0.06	0.03
Legumes	**0.35**	−0.06	0.02
Mushroom & fungi	**0.25**	0.07	−0.06
Fats and oils	−**0.39**	−0.20	0.11
Condiments	−0.06	**0.48**	−0.06
Coffee	−0.15	**0.41**	−0.15
Nuts	0.12	**0.39**	−0.03
Fast food	−0.02	**0.35**	0.14
French fries and potato chips	−0.02	**0.34**	0.14
Milk and milk products	0.11	**0.33**	−0.10
Whole grains	0.16	**0.32**	−0.16
Sweets and desserts	0.01	**0.27**	0.19
Beverages	−0.02	**0.22**	0.10
Eggs	0.08	**0.22**	0.05
Dim sum	−0.17	−0.17	**0.51**
Red and processed meats	−0.07	−0.03	**0.47**
Poultry	0.06	0.03	**0.47**
Fish and seafood	0.20	−0.26	**0.33**
Cakes, cookies, pies and biscuits	0.07	0.12	**0.24**
Wine	−0.15	0.07	**0.22**
Refined grains	−0.24	−0.42	−**0.74**
Organ meats	−0.08	0.13	0.12
Others	0.01	0.07	0.05
Preserved vegetables	0	0.10	0.01
Soups	0	0	−0.01
Tea	−0.03	0.17	0.05
**% variance explained**	**6.5**	**5.2**	**5.2**

a Factor loadings with absolute value ≥0.2 are shown in bold (Field 2005). For food group loads more than one dietary pattern, only the highest absolute value of loading is bolded.

‘Vegetables-fruits’ dietary pattern scores were inversely associated with total fat, saturated fat, monounsaturated fat and polyunsaturated fat intakes, and positively associated with intakes of protein, fiber, isoflavones, most vitamins and minerals. ‘Snacks-drinks-milk products’ dietary pattern scores were inversely associated with intakes of polyunsaturated fat and vitamin K, and positively associated with intakes of carbohydrates, protein, total fat, saturated fat, monounsaturated fat, cholesterol, fiber, most minerals and vitamins. Whilst ‘snacks-drinks-milk products’ dietary pattern scores were also positively associated with intakes of fiber and most minerals and vitamins, the associations were less strong than those with ‘vegetables-fruits’ dietary pattern scores. ‘Meat-fish’ dietary pattern scores were weakly positively associated with intakes of polyunsaturated fat and most micronutrients, moderately positively associated with intakes of protein, total fat and monounsaturated fat, and strongly positively associated with saturated fat and cholesterol intakes. The positive associations of protein, total fat, different kinds of fat and cholesterol with ‘meat-fish’ dietary pattern scores were stronger than those with ‘snacks-drinks-milk products’ dietary pattern scores (Table 2).

**Table 2:** Pearson's correlations between each dietary pattern score and nutrient intakes at baseline (n = 2,902).

Nutrients	Vegetables-fruits		Snacks-drinks-milk products		Meat-fish	
	r	*p*	r	*p*	r	*P*
Energy (kcal)	−0.05	0.006	0.21	<0.001	0.22	<0.001
Carbohydrates (g)	−0.01	0.456	0.17	<0.001	−0.11	<0.001
Protein (g)	0.13	<0.001	0.21	<0.001	0.39	<0.001
Fat (g)	−0.12	<0.001	0.18	<0.001	0.48	<0.001
SFA (% energy)	−0.17	<0.001	0.14	<0.001	0.57	<0.001
MUFA (% energy)	−0.06	0.001	0.06	0.001	0.36	<0.001
PUFA (% energy)	−0.13	<0.001	−0.11	<0.001	0.15	<0.001
Cholesterol (mg) †	0.03	0.168	0.15	<0.001	0.55	<0.001
Fiber (g) †	0.60	<0.001	0.17	<0.001	0.04	0.019
Vitamin A (IU) †	0.57	<0.001	0.05	0.006	0.11	<0.001
Vitamin C (mg) †	0.52	<0.001	0.03	0.103	0.12	<0.001
Calcium (mg)	0.36	<0.001	0.31	<0.001	0.02	0.258
Phosphorus (mg)	0.19	<0.001	0.33	<0.001	0.07	<0.001
Iron (mg) †	0.28	<0.001	0.26	<0.001	0.18	<0.001
Potassium (mg) †	0.24	<0.001	0.08	<0.001	0.33	<0.001
Magnesium (mg) †	0.38	<0.001	0.07	<0.001	−0.01	0.645
Sodium (mg) †	0.12	<0.001	0.23	<0.001	0.31	<0.001
Zinc (mg)	0.20	<0.001	0.15	<0.001	0.28	<0.001
Isoflavones (mg) †	0.35	<0.001	0.09	<0.001	0.13	<0.001
Vitamin K (µg) †	0.52	<0.001	−0.13	<0.001	0.07	<0.001
Vitamin D (IU) †	0.10	<0.001	0.30	<0.001	0.06	0.001

SFA, Saturated fatty acids; MUFA, Monounsaturated fatty acids; PUFA, Polyunsaturated fatty acids.

† log transformed nutrient intake.

### Participants' characteristics by baseline depressive status

Participants who were excluded in the cross-sectional analysis were older and less physically active, had lower education level, and were more likely to be female, widowed, separated, divorced or single, and to have self-reported history of diabetes than those who were included in the cross-sectional analysis (*p*<0.05). Those who discontinued the 4-year follow-up were older, less physically active, and were more likely to be divorced or single as compared to those who completed the 4-year follow-up (*p*<0.05) (details not shown).

Among 2,902 participants included in the cross-sectional analysis, there were 218 (7.5%) participants who were classified as having depressive symptoms at baseline. For the longitudinal analysis, 81 (3.7%) cases were newly identified as having depressive symptoms out of 2,211 participants at the 4-year follow-up. The characteristics of participants with depressive symptoms and participants without depressive symptoms at baseline are shown in Table 3. Those who had depressive symptoms had lower CSI-D score, lower education level, more impairments in IADLs, and were more likely to be widowed, separated, divorced or single, and current smokers, and were more likely to have self-reported history of heart diseases or stroke. Those who had depressive symptoms also showed lower ‘vegetables-fruits’ dietary pattern scores and lower ‘snacks-drinks-milk products’ dietary pattern scores.

**Table 3:** Subject characteristics by baseline depressive status (n = 2,902).

	Subjects without depressive symptoms (GDS<8) (n = 2,684)		Subjects with depressive symptoms (GDS> = 8) (n = 218)		*P value* 1
	Mean/n	(SD/%)	Mean/n	SD/%	
Age (years)	71.8	(4.8)	72.2	(5.0)	0.310
BMI (kg/m^2^)	23.6	(3.2)	23.4	(3.3)	0.203
PASE score	94.1	(44.7)	91.5	(40.2)	0.399
CSI-D score	31.2	(0.9)	31.0	(0.8)	**0.001**
Daily energy intake (kcal)	1913.8	(571.4)	1876.3	(556.7)	0.351
Dietary pattern scores					
Vegetables-fruits	0.015	(1.002)	−0.187	(0.958)	**0.004**
Q1	651	(24.3)	76	(34.9)	**0.002**
Q2	677	(25.2)	49	(22.5)	
Q3	669	(24.9)	55	(25.2)	
Q4	687	(25.6)	38	(17.4)	
Snacks-drinks-milk products	0.022	(1.004)	−0.272	(0.915)	**<0.001**
Q1	646	(24.1)	81	(37.2)	**<0.001**
Q2	662	(24.7)	62	(28.4)	
Q3	684	(25.5)	43	(19.7)	
Q4	692	(25.8)	32	(14.7)	
Meat-fish	0.004	(0.996)	−0.045	(1.051)	0.487
Q1	669	(24.9)	56	(25.7)	0.441
Q2	665	(24.8)	61	(28.0)	
Q3	670	(25.0)	56	(25.7)	
Q4	680	(25.3)	45	(20.6)	
Female (%)	1075	(40.1)	87	(39.9)	0.967
Education level (%)					
Primary or below	1697	(63.2)	157	(72.0)	**0.014**
Secondary / Matriculation	636	(23.7)	45	(20.6)	
University or above	351	(13.1)	16	(7.3)	
Marital status (%)					
Married or living with a partner	2085	(77.7)	155	(71.1)	**0.004**
Widowed, separated or divorced	546	(20.3)	52	(23.9)	
Single	53	(2.0)	11	(5.0)	
Smoking status (%)					
Never smoke	1583	(59.0)	110	(50.5)	**0.003**
Ex-smoker	910	(33.9)	80	(36.7)	
Current smoker	191	(7.1)	28	(12.8)	
Alcohol use (%)					
Never	2203	(82.1)	181	(83.0)	0.191
Ex-drinker	55	(2.0)	8	(3.7)	
Current drinker	426	(15.9)	29	(13.3)	
Self-reported medical history (%)					
Diabetes mellitus	350	(13.0)	38	(17.4)	0.067
Hypertension	1118	(41.7)	100	(45.9)	0.225
Heart diseases or stroke	552	(20.6)	71	(32.6)	**<0.001**
IADLs					
No impairment	2141	(79.8)	138	(63.3)	**<0.001**
Some impairments	543	(20.2)	80	(36.7)	

1 Differences between groups were assessed by independent *t* test or chi square test.

### Dietary patterns and the presence of depressive symptoms at baseline and 4-year

In the cross-sectional analysis, participants in the highest quartile of “vegetables-fruits” pattern score had reduced likelihood of depressive symptoms [Adjusted OR = 0.55 (95% CI: 0.36–0.83), *p_trend_* = 0.017] compared to participants in the lowest quartile. Participants in the highest quartile of “snacks-drinks-milk products” pattern score also showed reduced likelihood of depressive symptoms [Adjusted OR = 0.41 (95% CI: 0.26–0.65), *p_trend_*<0.001] compared to those in the lowest quartile. There was no association of “meat-fish” pattern with depressive status at baseline (Table 4). Longitudinal analysis showed that none of the dietary patterns were associated with the presence of depressive symptoms at 4-year (Table 5).

**Table 4:** Odds ratios (95% CI) for the cross-sectional association between dietary patterns and depressive symptoms (GDS> = 8) at baseline (n = 2,902).

Dietary pattern	Case / Control	Crude		*P_trend_* 1	Model 1^2^		*P_trend_*	Model 2^3^		*P_trend_*	Model 3^4^		*P_trend_*
		OR	(95% CI)		OR	(95% CI)		OR	(95% CI)		OR	(95% CI)	
Vegetables-fruits													
Q1	76/651	1		**0.001**	1		**0.001**	1		**0.019**	1		**0.017**
Q2	49/677	0.62	0.43–0.90		0.62	0.43–0.91		0.67	0.46–0.98		0.68	0.46–0.99	
Q3	55/669	0.70	0.49–1.01		0.71	0.49–1.02		0.81	0.56–1.18		0.83	0.57–1.20	
Q4	38/687	0.47	0.32–0.71		0.48	0.32–0.72		0.56	0.37–0.84		0.55	0.36–0.83	
Snacks- drinks-milk products													
Q1	81/646	1		**<0.001**	1		**<0.001**	1		**<0.001**	1		**<0.001**
Q2	62/662	0.75	0.53–1.06		0.75	0.53–1.06		0.76	0.53–1.09		0.77	0.54–1.10	
Q3	43/684	0.50	0.34–0.74		0.51	0.34–0.74		0.51	0.34–0.76		0.52	0.35–0.78	
Q4	32/692	0.37	0.24–0.56		0.37	0.24–0.57		0.40	0.25–0.62		0.41	0.26–0.65	
Meat-fish													
Q1	56/669	1		0.221	1		0.273	1		0.269	1		0.199
Q2	61/665	1.10	0.75–1.60		1.10	0.76–1.61		1.18	0.80–1.73		1.18	0.80–1.73	
Q3	56/670	1.00	0.68–1.47		1.01	0.69–1.49		1.08	0.73–1.61		1.06	0.71–1.58	
Q4	45/680	0.79	0.53–1.19		0.81	0.54–1.22		0.80	0.53–1.22		0.77	0.51–1.18	

1 Test for trend was examined by entering dietary pattern score quartiles as a fixed factor and testing the contrast by using the polynomial option in all models.

2 Model 1: adjusted for age, sex and daily energy intake.

3 Model 2: further adjusted for BMI, PASE, number of IADLs, smoking status, alcohol use, education and marital status.

4 Model 3: further adjusted for self-reported history of diabetes mellitus, hypertension, heart disease and stroke, and CSI-D score.

**Table 5:** Odds ratios (95% CI) for the longitudinal association between baseline dietary patterns and 4-year incidence of depressive symptoms (GDS> = 8) (n = 2,211).

Dietary pattern	Case / Control	Crude		*P_trend_* 1	Model 1^2^		*P_trend_*	Model 2^3^		*P_trend_*	Model 3^4^		*P_trend_*
		OR	(95% CI)		OR	(95% CI)		OR	(95% CI)		OR	(95% CI)	
Vegetables-fruits													
Q1	25/494	1		0.415	1		0.432	1		0.903	1		0.931
Q2	17/547	0.61	0.33–1.15		0.62	0.33–1.17		0.71	0.38–1.35		0.73	0.38–1.38	
Q3	18/534	0.67	0.36–1.24		0.66	0.36–1.24		0.77	0.41–1.45		0.80	0.42–1.50	
Q4	21/555	0.75	0.41–1.35		0.76	0.42–1.37		0.94	0.51–1.73		0.94	0.51–1.74	
Snacks- drinks-milk products													
Q1	24/494	1		0.430	1		0.569	1		0.845	1		0.824
Q2	13/514	0.52	0.26–1.03		0.54	0.27–1.07		0.61	0.31–1.21		0.62	0.31–1.24	
Q3	27/550	1.01	0.58–1.77		1.07	0.61–1.89		1.28	0.71–2.29		1.28	0.71–2.31	
Q4	17/572	0.61	0.33–1.15		0.65	0.34–1.24		0.84	0.43–1.65		0.85	0.43–1.68	
Meat-fish													
Q1	23/530	1		0.880	1		0.822	1		0.935	1		0.957
Q2	17/530	0.74	0.39–1.40		0.74	0.39–1.41		0.78	0.41–1.49		0.79	0.41–1.50	
Q3	16/533	0.69	0.36–1.32		0.71	0.37–1.36		0.74	0.38–1.44		0.75	0.39–1.45	
Q4	25/537	1.07	0.60–1.91		1.09	0.61–1.97		1.05	0.57–1.92		1.04	0.56–1.90	

1 Test for trend was examined by entering dietary pattern score quartiles as a fixed factor and testing the contrast by using the polynomial option in all models.

2 Model 1: adjusted for age, sex and daily energy intake at baseline.

3 Model 2: further adjusted for BMI, PASE, number of IADLs, smoking status, alcohol use, education and marital status at baseline.

4 Model 3: further adjusted for self-reported history of diabetes mellitus, hypertension, heart disease and stroke, and CSI-D score at baseline.

Sensitivity analysis excluding participants with extreme GDS (i.e. GDS≥11 (n = 56)) showed similar results for the cross-sectional data (details not shown). Multivariate logistic regression models that were repeated using a group with an increase in GDS score > = 4 over four years as an outcome also showed no association between each dietary pattern and the presence of depressive symptoms at 4-year (details not shown).

## Discussion

This study found an inverse association between “vegetables-fruits’ and ‘snacks-drinks-milk products’ dietary pattern scores and baseline depressive symptoms. However, no significant associations between ‘meat-fish’ dietary pattern scores and baseline depressive symptoms were observed. Moreover, we did not observe significant associations between baseline dietary pattern scores and subsequent depressive symptoms at 4-year.

Previous cross-sectional studies have evaluated the association between dietary patterns and depression or depressive symptoms. However, the results are inconclusive [13]–[16], [22]. Similar to our findings, two recent studies from Japan showed that a healthy Japanese dietary pattern characterized by high intakes of plant foods including vegetables, fruit, mushrooms and soy products was associated with fewer depressive symptoms [14], [16]. Our results were also consistent with the findings from an Australian study which showed that a diet characterized by high intakes of fruits, vegetables, and plant based foods was associated with fewer depressive symptoms[13]. Compared to the “snacks-drinks-milk products” pattern and the “meat-fish” pattern, the “vegetables-fruits” pattern showed the highest correlations with fiber and other nutrients, such as vitamin C that are considered as beneficial for brain health [6]. It is possible that the “vegetables-fruits” pattern is associated with reduced oxidative stress, higher anti-inflammatory property, and higher antioxidant capacity, the potential biological mechanisms that are linked with better mood and brain health [44]. The potential protective effect of the ‘vegetables-fruits” pattern could also come from folate that is rich in some cruciferous vegetables, leafy vegetables, and other green vegetables. There has been evidence to suggest that folate deficiency impairs the synthesis of homocysteine to methionine and S-adenosyl-methionine. The latter is a methyl donor and is involved in the synthesis and metabolism of neurotransmitters [45]. There is also substantial evidence from observational studies and randomized controlled trials to suggest that folate deficiency is associated with increased risk of depression [7], [46].

Our study showed that ‘snacks-drinks-milk products’ dietary pattern was linked to a fewer depressive symptoms. Such an inverse association was less easy to interpret. In comparison to the ‘vegetable-fruit’ dietary pattern, the positive association of this pattern with intakes of fiber and most minerals and vitamins was less strong. However, this pattern was composed of healthy and unhealthy food groups. This pattern was dominated by the intake of condiments, drinks, sweets and desserts, fast food, French fries, potato chips, nuts, milk and milk products, and whole grains. Past studies have examined the role of these food groups in depression [47]–[49]. Greater intake of high-calorie sweet foods and fast foods appeared to be associated with more depressive symptoms [47], [48]. In contrast, there is evidence to suggest that increased intake of milk and dairy products, in particular the low-fat milk and dairy products is associated with better brain function and mood [49].

Our longitudinal analysis did not show significant associations between baseline dietary pattern scores and subsequent depressive symptoms at 4-year. Sensitivity analysis by repeating the multivariate logistic regression models with an outcome of a group with an increase in GDS score > = 4 over four years also confirmed these negative findings. Furthermore, in order to investigate the relationship between diet and depression persistence at 4-year, we tried to repeat the multivariate logistic regression models for participants with depressive symptoms at baseline. No association between dietary patterns and the presence of depressive symptoms was observed at 4-year among this group of baseline depressive participants and these findings further confirmed our negative longitudinal findings. Several prospective studies have examined the role of dietary patterns and incident depression or depressive symptoms in adulthood [17]–[25], and the findings generally support the role of dietary patterns in the development of depression. Most studies reported significant associations between dietary patterns and incident depression or depressive symptoms [17]–[19], [21]–[25], but one recent study showed no clear associations between dietary patterns and depression incidence [20]. Using data of 3,486 men and women of the Whitehall II prospective cohort, Akbaraly and colleagues showed a higher odds of depressive symptoms with a “processed food pattern” which was heavily loaded by high intake of sweetened desserts, chocolates, fried food, processed meat, pies, refined grains, high-fat dairy products and condiments [18]. In the GAZEL cohort, Le Port et al. [19] reported that healthy pattern (characterized by vegetables consumption) were associated with fewer depressive symptoms in men and women, and traditional pattern (characterized by fish and fruit consumption) were associated with lower risk of incident depression in women. Furthermore, the highest quartile of low-fat, western, high snack and high fat-sweet diets in men and low-fat and high snack diets in women were associated with higher likelihood of depressive symptoms compared to the lowest quartile. However, recent findings among women in the Nurses' Health Study did not suggest clear associations between dietary Prudent and Western pattern scores and incident depression risk [20]. Differences in the study design, such as tools for assessing depression (i.e. self-reported scale vs. clinical diagnosis), inclusion and exclusion criteria for the subjects, and covariates that were included in the statistical models may account for the variations in the study findings [50]. Recent findings from a multicenter, randomized, primary prevention filed trial suggest that a Mediterranean diet supplemented with nuts could possibly reduce the risk of depression in patients with type 2 diabetes [51]. Therefore, the role of diet in depression warrants further investigation.

Strengths of our study are inclusion of both cross-sectional and longitudinal study design, and adjustment for several potential confounders. However, our study had several limitations. First, the use of the self-reported measure of depression (GDS) and cognitive impairment (CSI-D) may be one of the limitations. However, the sensitivity of these measures is high and has been validated in Chinese populations [32], [36]. Second, the low number of participants with depressive symptoms at the 4-year follow-up may have limited the power of the longitudinal analysis. Although we found significant associations between “vegetables-fruits” and “snacks-drinks-milk products” patterns and depressive symptoms in the cross-sectional analysis, the directionality of the associations was uncertain. The possibility of reverse causation may not be ruled out. People with major depression may change their eating behavior and food choices, either adopting an unhealthy diet (i.e. high-calorie foods) or reducing food intake [47], [52], thus interpreting the relationship between diet and depression may be difficult in view of the bidirectional changes in diet as a consequence of mental health symptoms. We tried to conduct a sensitivity analysis excluding participants with extreme GDS (i.e. GDS≥11 (n = 56)) and the significant association between dietary patterns and depressive symptoms remained at baseline. These results possibly suggest that reverse causation seems an unlikely explanation for the significant findings in our cross-sectional analysis. Furthermore, we did not have dietary data at the 4-year follow-up whereas dietary patterns may have changed between baseline and follow-up. Moreover, although we controlled for various common factors and major chronic conditions in the analysis, residual potential confounding from some other factors related to the development of depression, such as family history of depression and recent life stress events might still be present. In addition, our sample as a whole was of a higher educational standard compared with the general Hong Kong population, and there were some differences in the baseline characteristics between those who included and those who excluded in the analysis, and between those who completed and those who discontinued the 4-year follow-up. Therefore, the results may not be generalized to the general population.

In conclusion, our cross-sectional analysis suggested significant associations between ‘vegetables-fruits’ dietary pattern and ‘snacks-drinks-milk products’ dietary pattern and the presence of depressive symptoms in the community-dwelling Chinese older people in Hong Kong. However, the longitudinal analyses did not support that dietary patterns are predictive of the occurrence of depressive symptoms.
